# Editorial: Beyond gastrointestinal symptoms: psychosocial implications of disorders of gut-brain interaction.

**DOI:** 10.3389/fmed.2026.1965333

**Published:** 2026-09-01

**Authors:** Bryan Adrian Priego-Parra, Francisco Alejandro Felix-Tellez

**Affiliations:** 1 Laboratorio de Fisiología Digestiva y Motilidad Gastrointestinal, Instituto de Investigaciones Médico-Biológicas, Universidad Veracruzana, Veracruz, México; 2 Departamento de Gastroenterología, Facultad de Medicina y Nutrición, Universidad Autónoma de Baja California, Mexicali, México

**Keywords:** disorders of gut brain interaction, functional gastrointestinal disorders, irritable bowel syndrome, mental health, neuropsychiatric symptoms, psychogastroenterology

## Introduction

Modern gastroenterology is increasingly moving beyond an organ-centered view of disease. Advances in neuroscience, immunology, microbiome research, and behavioral science have shown that digestive disorders arise from the continuous interaction of biological, psychological, environmental, and sociocultural processes. This shift has been particularly evident in disorders of gut–brain interaction (DGBIs), in which altered motility, visceral hypersensitivity, immune activation, microbial changes, autonomic dysfunction, and disrupted gut–brain signaling interact with psychological and behavioral factors to shape symptom generation and disease burden. This multidimensional perspective is increasingly relevant across the spectrum of gastrointestinal diseases, including conditions traditionally regarded as purely organic (1). The recent introduction of Rome V (2), consolidates this conceptual evolution by further moving the field away from the label “functional gastrointestinal disorders” and reinforcing the term DGBIs (3). This terminology better reflects current pathophysiological understanding, supports greater diagnostic precision, and provides a shared language grounded in a patient-centered biopsychosocial model (4).

**Figure 1:**
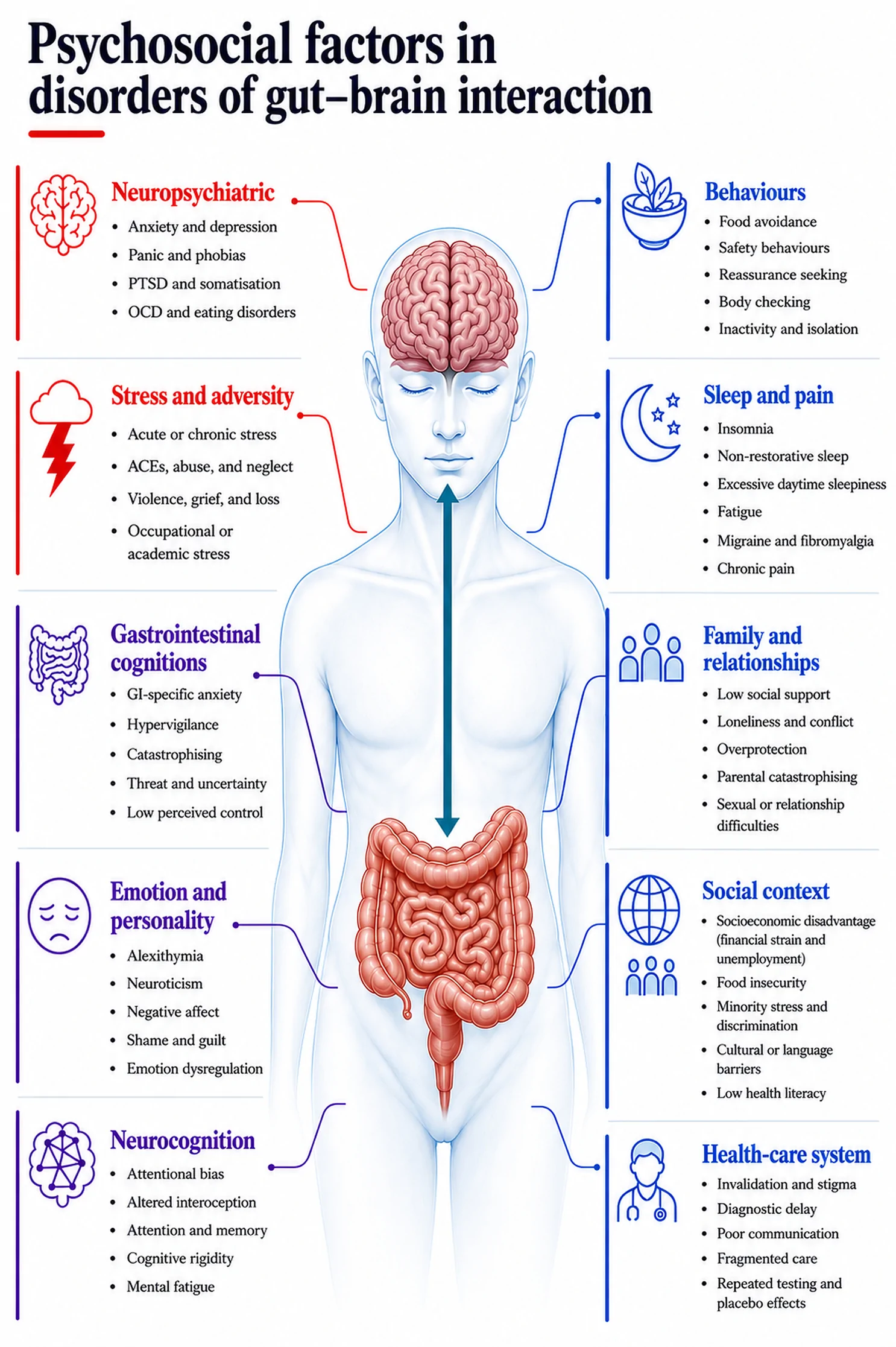
Psychosocial factors associated with disorders of gut–brain interaction. ACEs, adverse childhood experiences; GI, gastrointestinal; OCD, obsessive–compulsive disorder; PTSD, post-traumatic stress disorder.

A growing body of evidence demonstrates that factors including sexual or gender minority status (5), adverse childhood experiences (6, 7), chronic stress, anxiety, depression, neuroticism (8), maladaptive symptom-related cognitions such as hypervigilance and catastrophizing (9), influence not only symptom severity but also patients' daily lives. Reduced quality of life (QoL), lower happiness and life satisfaction, sexual dysfunction and urinary symptoms (10), disordered eating behaviors (11, 12), presenteeism, absenteeism (13) and impaired social functioning are increasingly recognized as part of the clinical burden of digestive disorders. Understanding these conditions therefore requires moving beyond pathophysiology alone to consider the individual's lived experience (Figure 1).

The studies presented in this research topic reflect this evolving vision of gastroenterology. Together, they span the life course and explore how biological, psychological, dietary, cultural, and sociocultural factors interact to shape digestive health. The narrative begins in early life with the investigation of umbilical cord biomarkers and their potential association with infantile colic and excessive crying. In an outstanding hypothesis article, Simonin et al. found that infants who later developed colic or excessive crying had higher trans fatty acid levels and a greater *Gammaproteobacteria* signature in cord blood at birth. This innovative study suggests that perinatal metabolic and microbial factors may contribute to the early development of DGBIs.

Complementing these mechanistic studies, Fu et al. and Xu et al. provided cross-culturally validated versions of the *Visceral Sensitivity Index* and the *Gastrointestinal Unhelpful Thinking Scale*, respectively, while Peng et al. examined sensory processing sensitivity as an individual trait among patients with DGBIs. Together, these studies strengthen the assessment of the psychological, cognitive, and perceptual dimensions associated with symptom experience and disease burden across diverse patient populations.

Irritable bowel syndrome (IBS) emerged as a recurring theme. Wang et al. reported elevated serum FGL2 levels, leukocyte and neutrophil counts, and proinflammatory cytokines in patients with IBS-D compared with healthy controls. FGL2 also showed strong discriminatory performance and was associated with inflammation, symptom severity, and psychological distress, highlighting its potential as a biomarker of disease burden. From another perspective, Rajabi and Ramdass found that individuals with myalgic encephalomyelitis/chronic fatigue syndrome had significantly greater odds of IBS than controls, suggesting possible shared underlying mechanisms. Complementing this, other studies highlighted the importance of modifiable lifestyle factors. Li et al. showed that greater consumption of ultra-processed foods was associated with an increased risk of IBS and functional dyspepsia (FD), whereas Altinsoy et al. found that mindful eating and adherence to a chrono-Mediterranean dietary pattern were associated with a lower burden of gastrointestinal symptoms.

This integrative perspective extends across a broader spectrum of digestive diseases. Volarić et al. explored the interplay between sex and emotional well-being in FD; Jiang et al. examined how somatic symptom disorder shapes symptom severity and QoL in gastro-esophageal reflux disease; meanwhile, Wang et al. are investigating vascular and autonomic correlates of depressive symptoms in chronic atrophic gastritis.

The issue also addresses clinical practice. Takekita et al. reported post-marketing data on elobixibat in individuals with chronic constipation and psychiatric comorbidities, while Arivalagan et al. described a case report of a paralytic ileus as an early manifestation of neuroleptic malignant syndrome, illustrating how gastrointestinal symptoms may herald a life-threatening systemic disorder. Finally, Liang et al. developed a predictive model to assess the association between acute gastrointestinal bleeding and cerebral infarction.

Rather than presenting digestive disorders as isolated entities, these studies collectively portray gastrointestinal health as the product of interconnected biological, psychological, behavioral, dietary, autonomic, cultural, and social influences. In doing so, they reflect the continuing evolution of gastroenterology towards a truly person-centered discipline, in which understanding the gut also requires understanding the brain, the environment, the mind and the lived experience of the individual.

## Future directions

The shift toward precision medicine and multimodal assessment is challenging traditional diagnostic approaches. DGBIs are complex because physiological changes and individual experiences interact to shape symptoms and disease burden. Cluster analyses have identified distinct patient profiles based on gastrointestinal and extraintestinal symptoms, psychological burden, lifestyle, and biological factors (14–19). Microbiome and multi-omics analyses may further refine these profiles and help explain why patients with the same Rome V diagnosis can have different symptoms and treatment responses. Although these tools are not yet ready for routine clinical use, they may support more personalized care in the future. Moreover, new hypotheses suggest that modern life may cause “sensory malnutrition” by reducing diverse body stimuli, disrupting body-brain communication and contributing to anxiety, somatic hypervigilance, visceral hypersensitivity and DGBIs, offering new perspectives for future research (20).

## Conclusion

As our understanding of digestive diseases moves beyond gastrointestinal symptoms alone, integrating biological and psychosocial mechanisms will be essential to explain clinical heterogeneity and advance precision medicine. Combining multi-omics approaches, artificial intelligence, microbiota analysis, and comprehensive clinical and psychological phenotyping may enable a deeper understanding of disease mechanisms and support more accurate prediction, prevention, and personalized management of DGBIs.

## References

[1] Elsenbruch S Ballou S Keefer L Murphy TB Van Oudenhove L Van Tilburg MAL. Biopsychosocial aspects of adult and pediatric disorders of gut-brain interaction. Gastroenterology. (2026) 170:1205–23. 10.1053/j.gastro.2026.02.009 42031440

[2] Drossman DA Chang L Tack J. Disorders of gut-brain interaction and the Rome V process. Gastroenterology. (2026) 170:1083–98. 10.1053/j.gastro.2026.02.014 42031435

[3] Remes-Troche JM. Rome V: minimizing “functional”, maximizing precision in gut-brain disorders. Rev Esp Enferm Dig. (2026) 118:438–41. 10.17235/reed.2026.12073/2026 PMID: 42439384 42439384

[4] Guadagnoli L Heathcote LC Van Oudenhove L Elsenbruch S Keefer L. The psychobiological model of disorders of gut-brain interaction: introduction of a novel, integrated, and testable model. Lancet Gastroenterol Hepatol. (2025) 10:1041–52. 10.1016/S2468-1253(25)00205-5 40886718

[5] Vélez C Casimiro I Pitts R Streed C Paul S. Digestive health in sexual and gender minority populations. Am J Gastroenterol. (2022) 117:865–75. 10.14309/ajg.0000000000001804 35537864

[6] Ren YT Xiao X Zhang K Chen XW Yu GP Fu W. Association between childhood trauma and the number of disorders of gut-brain interaction diagnosed in adults, as well as the mediating role of personality disorder traits and anxiety. Zhonghua Yi Xue Za Zhi. (2026) 106:650–7. 10.3760/cma.j.cn112137-20250707-01643 41688172

[7] Fritz J Coffey R Bloch J Cutler A Gabrielson S DiGiovanni S. The relationship between adverse childhood experiences and disorders of the gut-brain interaction. J Pediatr Gastroenterol Nutr. (2025) 80:100–7. 10.1002/jpn3.12422 39584227

[8] Ye W Ding H Peng B Wang X Cheng Y Zhang G. Large-scale meta- and cross-trait analyses uncover shared genetic risk factors for IBS and psychiatric disorders. Front Psychiatry. (2026) 17:1795532. 10.3389/fpsyt.2026.1795532 42221328 PMC13216503

[9] Priego-Parra BA Reyes-Diaz SA Ordaz-Alvarez HR Martínez-Pérez G-P Amieva-Balmori M García-Zermeño KR. Gastrointestinal cognition: pain catastrophizing in irritable bowel syndrome, a cross-sectional study in Mexico. Neurogastroenterol Motil. (2025) 37:e70022. 10.1111/nmo.70022 40125778

[10] Felix-Tellez FA Del Rio ÓBrien MF Ibarra Tapia ME Escobar Montes MA Peña Barajas GJ Mercado Basoco SA. Association between irritable bowel syndrome and lower urinary tract symptomatology: a cross-sectional study in Mexican population. J Clin Gastroenterol. (2025) 59:874–9. 10.1097/MCG.0000000000002093 39453697

[11] Ordaz-Alvarez HR Priego-Parra BA Reyes-Diaz SA Garcia-Zermeño KR Del Rocio Francisco M Amieva-Balmori M. Prevalence of eating disorders among adults with irritable bowel syndrome: a cross-sectional study. J Clin Gastroenterol. (2025) 59:880–8. 10.1097/MCG.0000000000002101 39918257

[12] Félix-Téllez FA Cruz-Salgado AX Remes-Troche JM Flores-Rendon ÁR Ordaz-Álvarez HR Velasco JAV-R. Association between functional dyspepsia and binge eating disorder: a frequent, often overlooked overlap clinical presentation. J Neurogastroenterol Motil. (2025) 31:95–101. 10.5056/jnm24070 39779207 PMC11735201

[13] Frändemark Å Törnblom H Hreinsson JP Andresen V Benninga MA Corazziari ES. Work productivity and activity impairment in disorders of gut-brain interaction: data from the Rome foundation global epidemiology study. United European Gastroenterol J. (2023) 11:503–13. 10.1002/ueg2.12425 PMC10337740 37332146

[14] Barberio B Pinto-Sanchez MI Bercik P Sood R Savarino EV Moayyedi P. Derivation and validation of a novel method to subgroup patients with functional dyspepsia: beyond upper gastrointestinal symptoms. Aliment Pharmacol Ther. (2021) 53:253–64. 10.1111/apt.16184 33280149

[15] Black CJ Yiannakou Y Guthrie EA West R Houghton LA Ford AC. A novel method to classify and subgroup patients with IBS based on gastrointestinal symptoms and psychological profiles. Am J Gastroenterol. (2021) 116:372–81. 10.14309/ajg.0000000000000975 33110014

[16] Black CJ Ng CE Goodoory VC Ford AC. Novel symptom subgroups in individuals with irritable bowel syndrome predict disease impact and burden. Clin Gastroenterol Hepatol. (2024) 22:386–396.e10. 10.1016/j.cgh.2023.02.016 36858142

[17] Colomier E Jones MP Holvoet L Van den Houte K Van de Bruaene C Broeders B. Symptom patterns outside the Rome IV consensus in eastern and western patients with a disorder of gut-brain interaction. Am J Gastroenterol. (2026). 10.14309/ajg.0000000000003996 41841607

[18] Balsiger LM Carbone F Raymenants K Scarpellini E Tack J. Understanding and managing patients with overlapping disorders of gut-brain interaction. Lancet Gastroenterol Hepatol. (2023) 8:383–90. 10.1016/S2468-1253(22)00435-6 36702144 PMC7615746

[19] Dowrick JM Roy NC Carco C James SC Heenan PE Frampton CMA. Integrated multi-omic and symptom clustering reveals lower-gastrointestinal disorders of gut-brain interaction heterogeneity. Gut Microbes. (2026) 18:2604871. 10.1080/19490976.2025.2604871 41431864 PMC12758187

[20] Bertuccioli A Palazzi CM Zonzini GB Belli A Pierro FD Cardinali M. Embodied sensory deprivation hypothesis (ESDH): an evolutionary model of sensory malnutrition linking interoceptive dysregulation, anxiety, and disorders of gut-brain interaction. Cureus. (2026) 18:e113925. 10.7759/cureus.113925 42553519 PMC13435178

